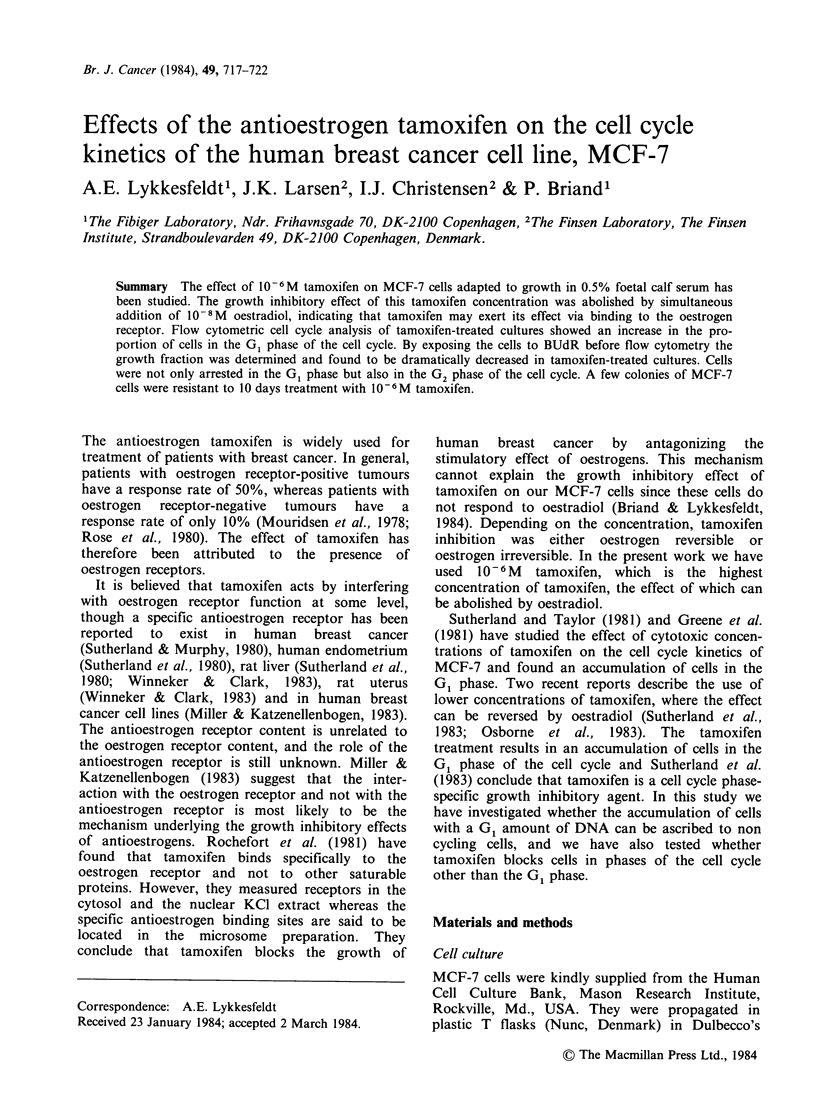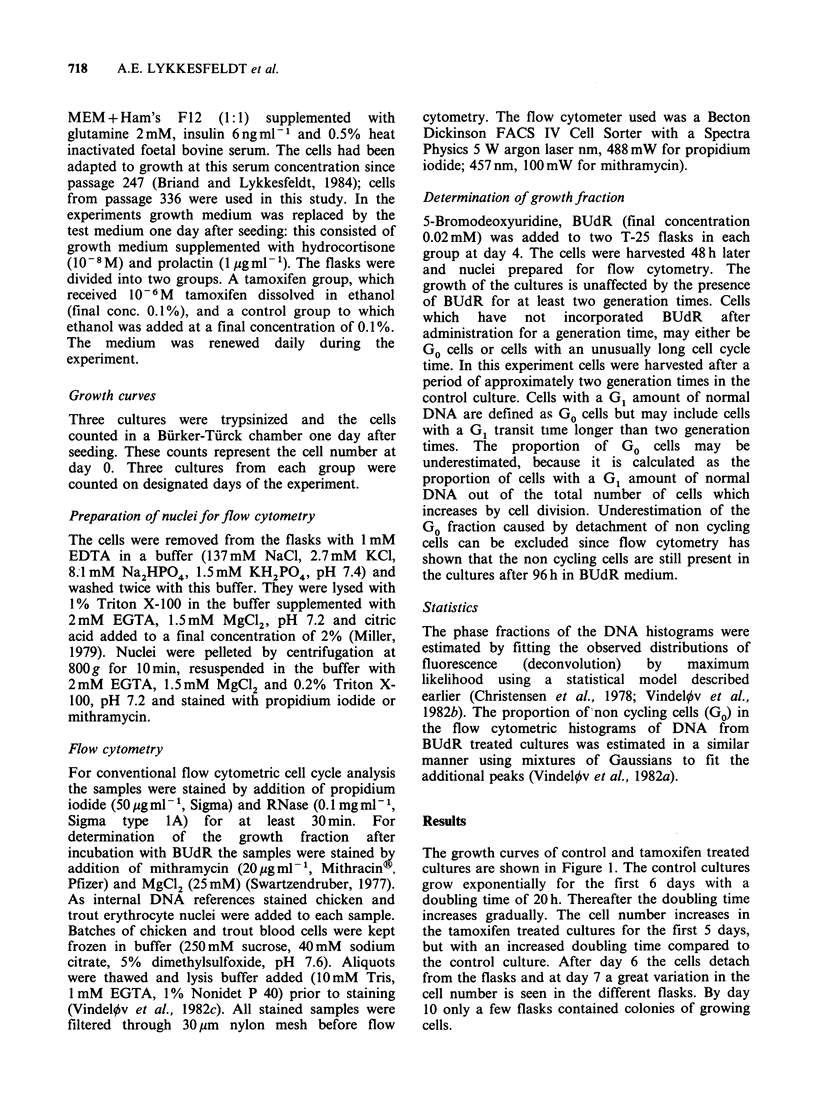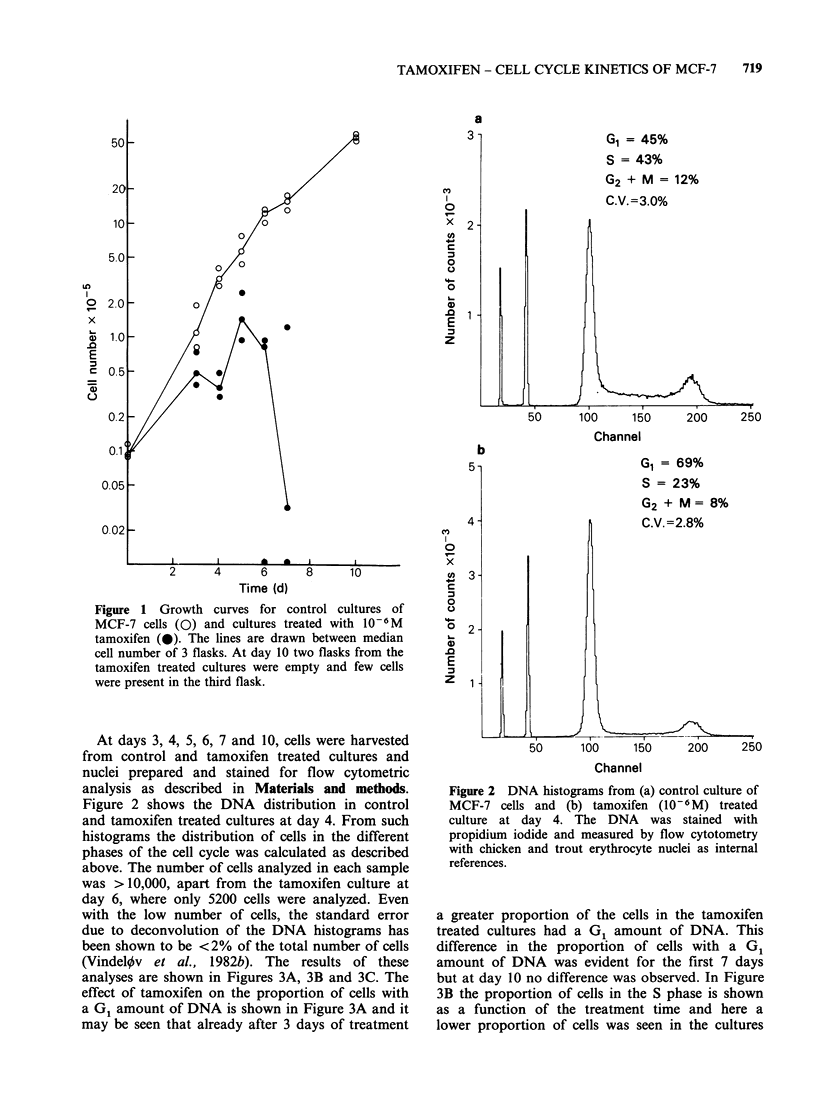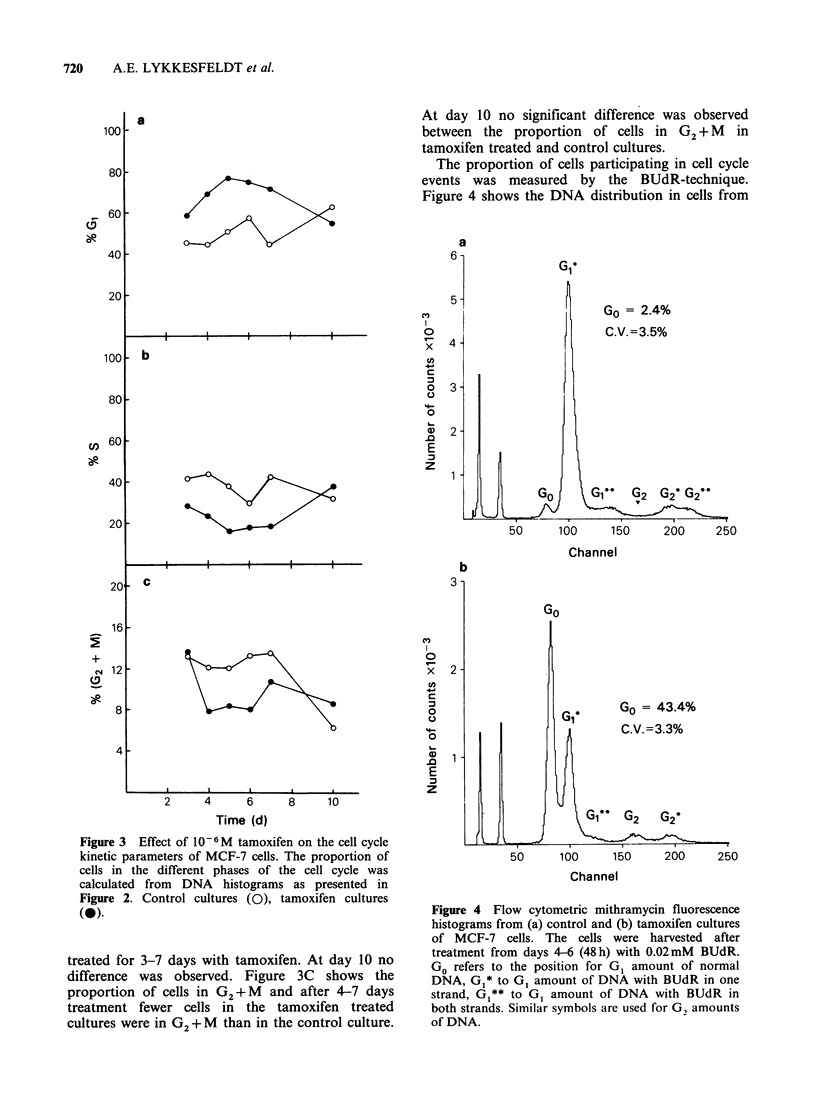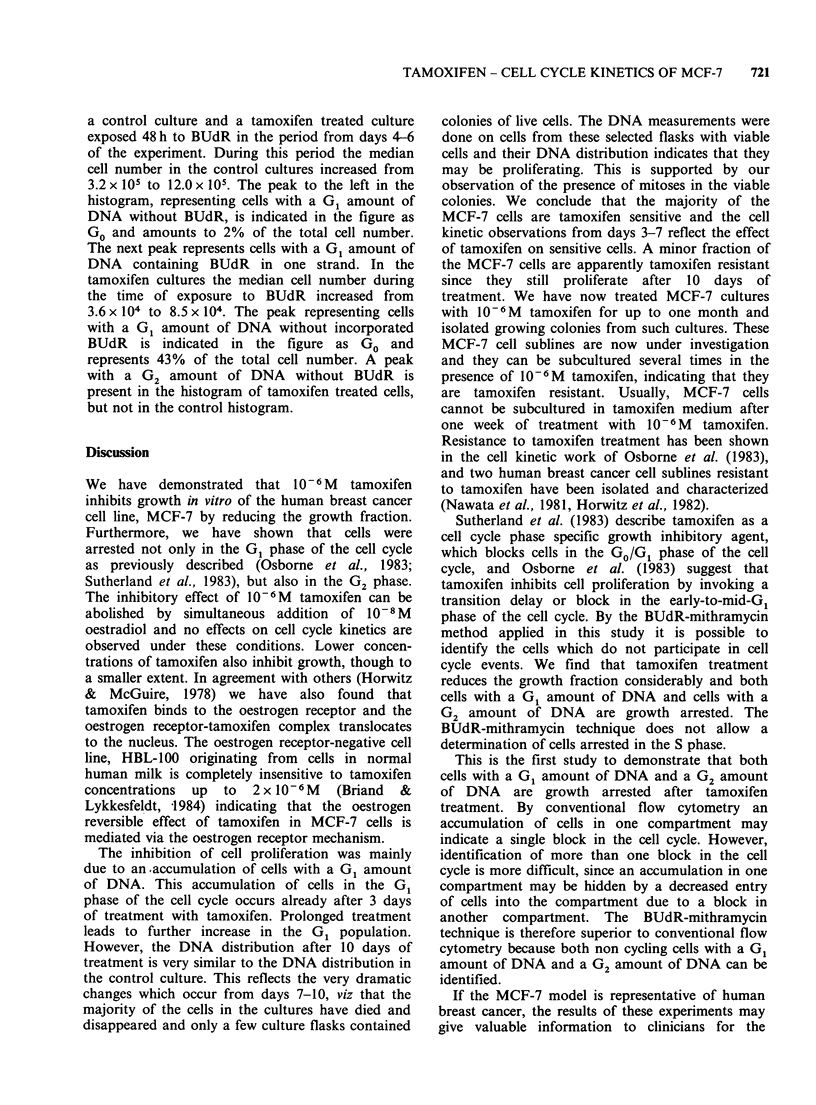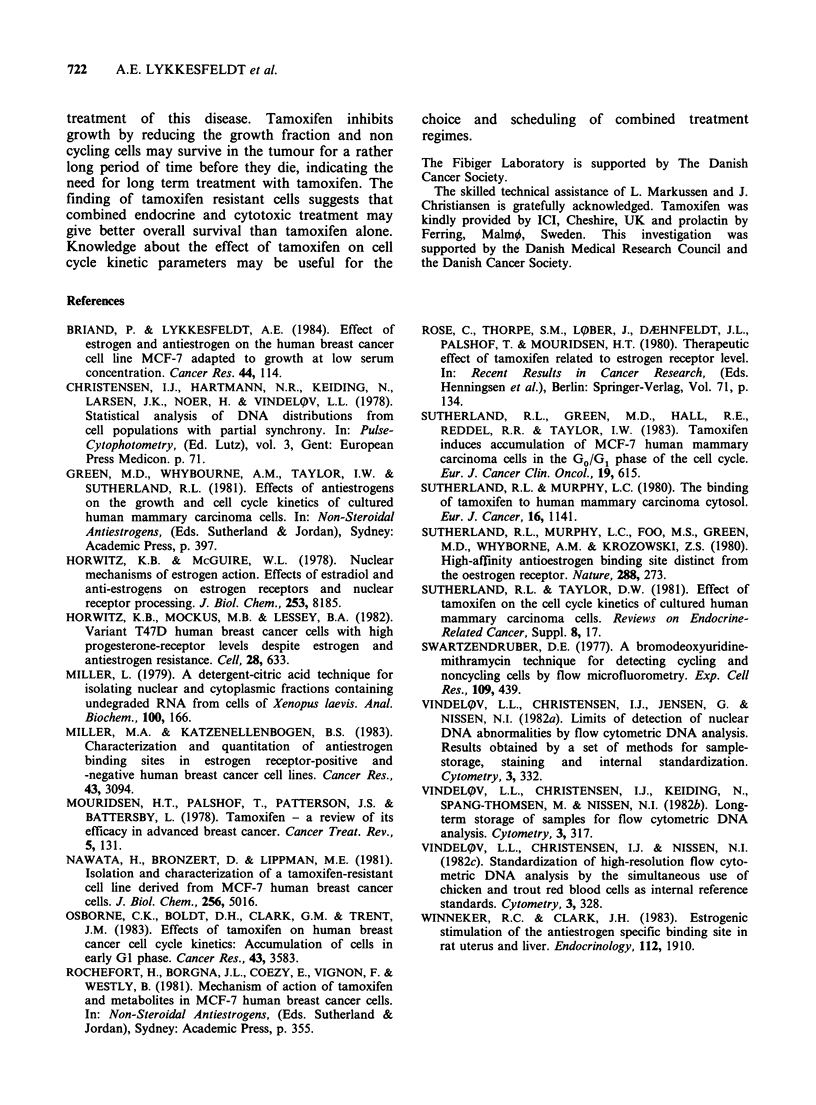# Effects of the antioestrogen tamoxifen on the cell cycle kinetics of the human breast cancer cell line, MCF-7.

**DOI:** 10.1038/bjc.1984.113

**Published:** 1984-06

**Authors:** A. E. Lykkesfeldt, J. K. Larsen, I. J. Christensen, P. Briand

## Abstract

The effect of 10(-6) M tamoxifen on MCF-7 cells adapted to growth in 0.5% foetal calf serum has been studied. The growth inhibitory effect of this tamoxifen concentration was abolished by simultaneous addition of 10(-8) M oestradiol, indicating that tamoxifen may exert its effect via binding to the oestrogen receptor. Flow cytometric cell cycle analysis of tamoxifen-treated cultures showed an increase in the proportion of cells in the G1 phase of the cell cycle. By exposing the cells to BUdR before flow cytometry the growth fraction was determined and found to be dramatically decreased in tamoxifen-treated cultures. Cells were not only arrested in the G1 phase but also in the G2 phase of the cell cycle. A few colonies of MCF-7 cells were resistant to 10 days treatment with 10(-6) M tamoxifen.


*Br. J. Cancer* (1984), **49**, 717–722

### Effects of the antioestrogen tamoxifen on the cell cycle kinetics of the human breast cancer cell line, MCF-7

A.E. Lykkesfeldt[1], J.K. Larsen[2], I.J. Christensen[2] & P. Briand[1]

[1]*The Fibiger Laboratory, Ndr. Frihavnsgade 70, DK-2100 Copenhagen,* [2]*The Finsen Laboratory, The Finsen Institute, Strandboulevarden 49, DK-2100 Copenhagen, Denmark.*

**Summary** The effect of $10^{-6}$M tamoxifen on MCF-7 cells adapted to growth in 0.5% foetal calf serum has been studied. The growth inhibitory effect of this tamoxifen concentration was abolished by simultaneous addition of $10^{-8}$M oestradiol, indicating that tamoxifen may exert its effect via binding to the oestrogen receptor. Flow cytometric cell cycle analysis of tamoxifen-treated cultures showed an increase in the proportion of cells in the $G_1$ phase of the cell cycle. By exposing the cells to BUdR before flow cytometry the growth fraction was determined and found to be dramatically decreased in tamoxifen-treated cultures. Cells were not only arrested in the $G_1$ phase but also in the $G_2$ phase of the cell cycle. A few colonies of MCF-7 cells were resistant to 10 days treatment with $10^{-6}$M tamoxifen.

The antioestrogen tamoxifen is widely used for treatment of patients with breast cancer. In general, patients with oestrogen receptor-positive tumours have a response rate of 50%, whereas patients with oestrogen receptor-negative tumours have a response rate of only 10% (Mouridsen *et al.*, 1978; Rose *et al.*, 1980). The effect of tamoxifen has therefore been attributed to the presence of oestrogen receptors.

It is believed that tamoxifen acts by interfering with oestrogen receptor function at some level, though a specific antioestrogen receptor has been reported to exist in human breast cancer (Sutherland & Murphy, 1980), human endometrium (Sutherland *et al.*, 1980), rat liver (Sutherland *et al.*, 1980; Winneker & Clark, 1983), rat uterus (Winneker & Clark, 1983) and in human breast cancer cell lines (Miller & Katzenellenbogen, 1983). The antioestrogen receptor content is unrelated to the oestrogen receptor content, and the role of the antioestrogen receptor is still unknown. Miller & Katzenellenbogen (1983) suggest that the interaction with the oestrogen receptor and not with the antioestrogen receptor is most likely to be the mechanism underlying the growth inhibitory effects of antioestrogens. Rochefort *et al.* (1981) have found that tamoxifen binds specifically to the oestrogen receptor and not to other saturable proteins. However, they measured receptors in the cytosol and the nuclear KCl extract whereas the specific antioestrogen binding sites are said to be located in the microsome preparation. They conclude that tamoxifen blocks the growth of human breast cancer by antagonizing the stimulatory effect of oestrogens. This mechanism cannot explain the growth inhibitory effect of tamoxifen on our MCF-7 cells since these cells do not respond to oestradiol (Briand & Lykkesfeldt, 1984). Depending on the concentration, tamoxifen inhibition was either oestrogen reversible or oestrogen irreversible. In the present work we have used $10^{-6}$M tamoxifen, which is the highest concentration of tamoxifen, the effect of which can be abolished by oestradiol.

Sutherland and Taylor (1981) and Greene *et al.* (1981) have studied the effect of cytotoxic concentrations of tamoxifen on the cell cycle kinetics of MCF-7 and found an accumulation of cells in the $G_1$ phase. Two recent reports describe the use of lower concentrations of tamoxifen, where the effect can be reversed by oestradiol (Sutherland *et al.*, 1983; Osborne *et al.*, 1983). The tamoxifen treatment results in an accumulation of cells in the $G_1$ phase of the cell cycle and Sutherland *et al.* (1983) conclude that tamoxifen is a cell cycle phase-specific growth inhibitory agent. In this study we have investigated whether the accumulation of cells with a $G_1$ amount of DNA can be ascribed to non cycling cells, and we have also tested whether tamoxifen blocks cells in phases of the cell cycle other than the $G_1$ phase.

### Materials and methods

### *Cell culture*

MCF-7 cells were kindly supplied from the Human Cell Culture Bank, Mason Research Institute, Rockville, Md., USA. They were propagated in plastic T flasks (Nunc, Denmark) in Dulbecco's

Correspondence: A.E. Lykkesfeldt
Received 23 January 1984; accepted 2 March 1984.

© The Macmillan Press Ltd., 1984

MEM+Ham's F12 (1:1) supplemented with glutamine 2 mM, insulin 6 ng ml$^{-1}$ and 0.5% heat inactivated foetal bovine serum. The cells had been adapted to growth at this serum concentration since passage 247 (Briand and Lykkesfeldt, 1984); cells from passage 336 were used in this study. In the experiments growth medium was replaced by the test medium one day after seeding: this consisted of growth medium supplemented with hydrocortisone ($10^{-8}$ M) and prolactin (1 $\mu$g ml$^{-1}$). The flasks were divided into two groups. A tamoxifen group, which received $10^{-6}$ M tamoxifen dissolved in ethanol (final conc. 0.1%), and a control group to which ethanol was added at a final concentration of 0.1%. The medium was renewed daily during the experiment.

*Growth curves*

Three cultures were trypsinized and the cells counted in a Bürker-Türck chamber one day after seeding. These counts represent the cell number at day 0. Three cultures from each group were counted on designated days of the experiment.

*Preparation of nuclei for flow cytometry*

The cells were removed from the flasks with 1 mM EDTA in a buffer (137 mM NaCl, 2.7 mM KCl, 8.1 mM $Na_2HPO_4$, 1.5 mM $KH_2PO_4$, pH 7.4) and washed twice with this buffer. They were lysed with 1% Triton X-100 in the buffer supplemented with 2 mM EGTA, 1.5 mM $MgCl_2$, pH 7.2 and citric acid added to a final concentration of 2% (Miller, 1979). Nuclei were pelleted by centrifugation at 800 *g* for 10 min, resuspended in the buffer with 2 mM EGTA, 1.5 mM $MgCl_2$ and 0.2% Triton X-100, pH 7.2 and stained with propidium iodide or mithramycin.

*Flow cytometry*

For conventional flow cytometric cell cycle analysis the samples were stained by addition of propidium iodide (50 $\mu$g ml$^{-1}$, Sigma) and RNase (0.1 mg ml$^{-1}$, Sigma type 1A) for at least 30 min. For determination of the growth fraction after incubation with BUdR the samples were stained by addition of mithramycin (20 $\mu$g ml$^{-1}$, Mithracin®, Pfizer) and $MgCl_2$ (25 mM) (Swartzendruber, 1977). As internal DNA references stained chicken and trout erythrocyte nuclei were added to each sample. Batches of chicken and trout blood cells were kept frozen in buffer (250 mM sucrose, 40 mM sodium citrate, 5% dimethylsulfoxide, pH 7.6). Aliquots were thawed and lysis buffer added (10 mM Tris, 1 mM EGTA, 1% Nonidet P 40) prior to staining (Vindeløv *et al.*, 1982*c*). All stained samples were filtered through 30 $\mu$m nylon mesh before flow cytometry. The flow cytometer used was a Becton Dickinson FACS IV Cell Sorter with a Spectra Physics 5 W argon laser nm, 488 mW for propidium iodide; 457 nm, 100 mW for mithramycin).

*Determination of growth fraction*

5-Bromodeoxyuridine, BUdR (final concentration 0.02 mM) was added to two T-25 flasks in each group at day 4. The cells were harvested 48 h later and nuclei prepared for flow cytometry. The growth of the cultures is unaffected by the presence of BUdR for at least two generation times. Cells which have not incorporated BUdR after administration for a generation time, may either be $G_0$ cells or cells with an unusually long cell cycle time. In this experiment cells were harvested after a period of approximately two generation times in the control culture. Cells with a $G_1$ amount of normal DNA are defined as $G_0$ cells but may include cells with a $G_1$ transit time longer than two generation times. The proportion of $G_0$ cells may be underestimated, because it is calculated as the proportion of cells with a $G_1$ amount of normal DNA out of the total number of cells which increases by cell division. Underestimation of the $G_0$ fraction caused by detachment of non cycling cells can be excluded since flow cytometry has shown that the non cycling cells are still present in the cultures after 96 h in BUdR medium.

*Statistics*

The phase fractions of the DNA histograms were estimated by fitting the observed distributions of fluorescence (deconvolution) by maximum likelihood using a statistical model described earlier (Christensen *et al.*, 1978; Vindeløv *et al.*, 1982*b*). The proportion of non cycling cells ($G_0$) in the flow cytometric histograms of DNA from BUdR treated cultures was estimated in a similar manner using mixtures of Gaussians to fit the additional peaks (Vindeløv *et al.*, 1982*a*).

### Results

The growth curves of control and tamoxifen treated cultures are shown in Figure 1. The control cultures grow exponentially for the first 6 days with a doubling time of 20 h. Thereafter the doubling time increases gradually. The cell number increases in the tamoxifen treated cultures for the first 5 days, but with an increased doubling time compared to the control culture. After day 6 the cells detach from the flasks and at day 7 a great variation in the cell number is seen in the different flasks. By day 10 only a few flasks contained colonies of growing cells.

**Figure 1** Growth curves for control cultures of MCF-7 cells (○) and cultures treated with $10^{-6}$M tamoxifen (●). The lines are drawn between median cell number of 3 flasks. At day 10 two flasks from the tamoxifen treated cultures were empty and few cells were present in the third flask.

**Figure 2** DNA histograms from (a) control culture of MCF-7 cells and (b) tamoxifen ($10^{-6}$M) treated culture at day 4. The DNA was stained with propidium iodide and measured by flow cytotometry with chicken and trout erythrocyte nuclei as internal references.

At days 3, 4, 5, 6, 7 and 10, cells were harvested from control and tamoxifen treated cultures and nuclei prepared and stained for flow cytometric analysis as described in **Materials and methods**. Figure 2 shows the DNA distribution in control and tamoxifen treated cultures at day 4. From such histograms the distribution of cells in the different phases of the cell cycle was calculated as described above. The number of cells analyzed in each sample was $>$10,000, apart from the tamoxifen culture at day 6, where only 5200 cells were analyzed. Even with the low number of cells, the standard error due to deconvolution of the DNA histograms has been shown to be $<$2% of the total number of cells (Vindeløv *et al.*, 1982*b*). The results of these analyses are shown in Figures 3A, 3B and 3C. The effect of tamoxifen on the proportion of cells with a $G_1$ amount of DNA is shown in Figure 3A and it may be seen that already after 3 days of treatment a greater proportion of the cells in the tamoxifen treated cultures had a $G_1$ amount of DNA. This difference in the proportion of cells with a $G_1$ amount of DNA was evident for the first 7 days but at day 10 no difference was observed. In Figure 3B the proportion of cells in the S phase is shown as a function of the treatment time and here a lower proportion of cells was seen in the cultures

**Figure 3** Effect of $10^{-6}$ M tamoxifen on the cell cycle kinetic parameters of MCF-7 cells. The proportion of cells in the different phases of the cell cycle was calculated from DNA histograms as presented in Figure 2. Control cultures (○), tamoxifen cultures (●).

treated for 3–7 days with tamoxifen. At day 10 no difference was observed. Figure 3C shows the proportion of cells in $G_2$+M and after 4–7 days treatment fewer cells in the tamoxifen treated cultures were in $G_2$+M than in the control culture.

At day 10 no significant difference was observed between the proportion of cells in $G_2$+M in tamoxifen treated and control cultures.

The proportion of cells participating in cell cycle events was measured by the BUdR-technique. Figure 4 shows the DNA distribution in cells from

**Figure 4** Flow cytometric mithramycin fluorescence histograms from (a) control and (b) tamoxifen cultures of MCF-7 cells. The cells were harvested after treatment from days 4–6 (48 h) with 0.02 mM BUdR. $G_0$ refers to the position for $G_1$ amount of normal DNA, $G_1$* to $G_1$ amount of DNA with BUdR in one strand, $G_1$** to $G_1$ amount of DNA with BUdR in both strands. Similar symbols are used for $G_2$ amounts of DNA.

a control culture and a tamoxifen treated culture exposed 48 h to BUdR in the period from days 4–6 of the experiment. During this period the median cell number in the control cultures increased from $3.2 \times 10^5$ to $12.0 \times 10^5$. The peak to the left in the histogram, representing cells with a $G_1$ amount of DNA without BUdR, is indicated in the figure as $G_0$ and amounts to 2% of the total cell number. The next peak represents cells with a $G_1$ amount of DNA containing BUdR in one strand. In the tamoxifen cultures the median cell number during the time of exposure to BUdR increased from $3.6 \times 10^4$ to $8.5 \times 10^4$. The peak representing cells with a $G_1$ amount of DNA without incorporated BUdR is indicated in the figure as $G_0$ and represents 43% of the total cell number. A peak with a $G_2$ amount of DNA without BUdR is present in the histogram of tamoxifen treated cells, but not in the control histogram.

### Discussion

We have demonstrated that $10^{-6}$ M tamoxifen inhibits growth *in vitro* of the human breast cancer cell line, MCF-7 by reducing the growth fraction. Furthermore, we have shown that cells were arrested not only in the $G_1$ phase of the cell cycle as previously described (Osborne *et al.*, 1983; Sutherland *et al.*, 1983), but also in the $G_2$ phase. The inhibitory effect of $10^{-6}$ M tamoxifen can be abolished by simultaneous addition of $10^{-8}$ M oestradiol and no effects on cell cycle kinetics are observed under these conditions. Lower concentrations of tamoxifen also inhibit growth, though to a smaller extent. In agreement with others (Horwitz & McGuire, 1978) we have also found that tamoxifen binds to the oestrogen receptor and the oestrogen receptor-tamoxifen complex translocates to the nucleus. The oestrogen receptor-negative cell line, HBL-100 originating from cells in normal human milk is completely insensitive to tamoxifen concentrations up to $2 \times 10^{-6}$ M (Briand & Lykkesfeldt, 1984) indicating that the oestrogen reversible effect of tamoxifen in MCF-7 cells is mediated via the oestrogen receptor mechanism.

The inhibition of cell proliferation was mainly due to an accumulation of cells with a $G_1$ amount of DNA. This accumulation of cells in the $G_1$ phase of the cell cycle occurs already after 3 days of treatment with tamoxifen. Prolonged treatment leads to further increase in the $G_1$ population. However, the DNA distribution after 10 days of treatment is very similar to the DNA distribution in the control culture. This reflects the very dramatic changes which occur from days 7–10, *viz* that the majority of the cells in the cultures have died and disappeared and only a few culture flasks contained colonies of live cells. The DNA measurements were done on cells from these selected flasks with viable cells and their DNA distribution indicates that they may be proliferating. This is supported by our observation of the presence of mitoses in the viable colonies. We conclude that the majority of the MCF-7 cells are tamoxifen sensitive and the cell kinetic observations from days 3–7 reflect the effect of tamoxifen on sensitive cells. A minor fraction of the MCF-7 cells are apparently tamoxifen resistant since they still proliferate after 10 days of treatment. We have now treated MCF-7 cultures with $10^{-6}$ M tamoxifen for up to one month and isolated growing colonies from such cultures. These MCF-7 cell sublines are now under investigation and they can be subcultured several times in the presence of $10^{-6}$ M tamoxifen, indicating that they are tamoxifen resistant. Usually, MCF-7 cells cannot be subcultured in tamoxifen medium after one week of treatment with $10^{-6}$ M tamoxifen. Resistance to tamoxifen treatment has been shown in the cell kinetic work of Osborne *et al.* (1983), and two human breast cancer cell sublines resistant to tamoxifen have been isolated and characterized (Nawata *et al.*, 1981, Horwitz *et al.*, 1982).

Sutherland *et al.* (1983) describe tamoxifen as a cell cycle phase specific growth inhibitory agent, which blocks cells in the $G_0/G_1$ phase of the cell cycle, and Osborne *et al.* (1983) suggest that tamoxifen inhibits cell proliferation by invoking a transition delay or block in the early-to-mid-$G_1$ phase of the cell cycle. By the BUdR-mithramycin method applied in this study it is possible to identify the cells which do not participate in cell cycle events. We find that tamoxifen treatment reduces the growth fraction considerably and both cells with a $G_1$ amount of DNA and cells with a $G_2$ amount of DNA are growth arrested. The BUdR-mithramycin technique does not allow a determination of cells arrested in the S phase.

This is the first study to demonstrate that both cells with a $G_1$ amount of DNA and a $G_2$ amount of DNA are growth arrested after tamoxifen treatment. By conventional flow cytometry an accumulation of cells in one compartment may indicate a single block in the cell cycle. However, identification of more than one block in the cell cycle is more difficult, since an accumulation in one compartment may be hidden by a decreased entry of cells into the compartment due to a block in another compartment. The BUdR-mithramycin technique is therefore superior to conventional flow cytometry because both non cycling cells with a $G_1$ amount of DNA and a $G_2$ amount of DNA can be identified.

If the MCF-7 model is representative of human breast cancer, the results of these experiments may give valuable information to clinicians for the treatment of this disease. Tamoxifen inhibits growth by reducing the growth fraction and non cycling cells may survive in the tumour for a rather long period of time before they die, indicating the need for long term treatment with tamoxifen. The finding of tamoxifen resistant cells suggests that combined endocrine and cytotoxic treatment may give better overall survival than tamoxifen alone. Knowledge about the effect of tamoxifen on cell cycle kinetic parameters may be useful for the choice and scheduling of combined treatment regimes.

The Fibiger Laboratory is supported by The Danish Cancer Society.

The skilled technical assistance of L. Markussen and J. Christiansen is gratefully acknowledged. Tamoxifen was kindly provided by ICI, Cheshire, UK and prolactin by Ferring, Malmφ, Sweden. This investigation was supported by the Danish Medical Research Council and the Danish Cancer Society.

### References

BRIAND, P. & LYKKESFELDT, A.E. (1984). Effect of estrogen and antiestrogen on the human breast cancer cell line MCF-7 adapted to growth at low serum concentration. *Cancer Res.* **44**, 114.

CHRISTENSEN, I.J., HARTMANN, N.R., KEIDING, N., LARSEN, J.K., NOER, H. & VINDELΦV, L.L. (1978). Statistical analysis of DNA distributions from cell populations with partial synchrony. In: *Pulse-Cytophotometry,* (Ed. Lutz), vol. 3, Gent: European Press Medicon. p. 71.

GREEN, M.D., WHYBOURNE, A.M., TAYLOR, I.W. & SUTHERLAND, R.L. (1981). Effects of antiestrogens on the growth and cell cycle kinetics of cultured human mammary carcinoma cells. In: *Non-Steroidal Antiestrogens,* (Eds. Sutherland & Jordan), Sydney: Academic Press, p. 397.

HORWITZ, K.B. & McGUIRE, W.L. (1978). Nuclear mechanisms of estrogen action. Effects of estradiol and anti-estrogens on estrogen receptors and nuclear receptor processing. *J. Biol. Chem.,* **253,** 8185.

HORWITZ, K.B., MOCKUS, M.B. & LESSEY, B.A. (1982). Variant T47D human breast cancer cells with high progesterone-receptor levels despite estrogen and antiestrogen resistance. *Cell,* **28,** 633.

MILLER, L. (1979). A detergent-citric acid technique for isolating nuclear and cytoplasmic fractions containing undegraded RNA from cells of *Xenopus laevis*. *Anal. Biochem.,* **100,** 166.

MILLER, M.A. & KATZENELLENBOGEN, B.S. (1983). Characterization and quantitation of antiestrogen binding sites in estrogen receptor-positive and -negative human breast cancer cell lines. *Cancer Res.,* **43,** 3094.

MOURIDSEN, H.T., PALSHOF, T., PATTERSON, J.S. & BATTERSBY, L. (1978). Tamoxifen – a review of its efficacy in advanced breast cancer. *Cancer Treat. Rev.,* **5,** 131.

NAWATA, H., BRONZERT, D. & LIPPMAN, M.E. (1981). Isolation and characterization of a tamoxifen-resistant cell line derived from MCF-7 human breast cancer cells. *J. Biol. Chem.,* **256,** 5016.

OSBORNE, C.K., BOLDT, D.H., CLARK, G.M. & TRENT, J.M. (1983). Effects of tamoxifen on human breast cancer cell cycle kinetics: Accumulation of cells in early G1 phase. *Cancer Res.,* **43,** 3583.

ROCHEFORT, H., BORGNA, J.L., COEZY, E., VIGNON, F. & WESTLY, B. (1981). Mechanism of action of tamoxifen and metabolites in MCF-7 human breast cancer cells. In: *Non-Steroidal Antiestrogens,* (Eds. Sutherland & Jordan), Sydney: Academic Press, p. 355.

ROSE, C., THORPE, S.M., LΦBER, J., DÆHNFELDT, J.L., PALSHOF, T. & MOURIDSEN, H.T. (1980). Therapeutic effect of tamoxifen related to estrogen receptor level. In: *Recent Results in Cancer Research,* (Eds. Henningsen *et al.*), Berlin: Springer-Verlag, Vol. 71, p. 134.

SUTHERLAND, R.L., GREEN, M.D., HALL, R.E., REDDEL, R.R. & TAYLOR, I.W. (1983). Tamoxifen induces accumulation of MCF-7 human mammary carcinoma cells in the $G_0/G_1$ phase of the cell cycle. *Eur. J. Cancer Clin. Oncol.,* **19,** 615.

SUTHERLAND, R.L. & MURPHY, L.C. (1980). The binding of tamoxifen to human mammary carcinoma cytosol. *Eur. J. Cancer,* **16,** 1141.

SUTHERLAND, R.L., MURPHY, L.C., FOO, M.S., GREEN, M.D., WHYBORNE, A.M. & KROZOWSKI, Z.S. (1980). High-affinity antioestrogen binding site distinct from the oestrogen receptor. *Nature,* **288,** 273.

SUTHERLAND, R.L. & TAYLOR, D.W. (1981). Effect of tamoxifen on the cell cycle kinetics of cultured human mammary carcinoma cells. *Reviews on Endocrine-Related Cancer,* Suppl. **8,** 17.

SWARTZENDRUBER, D.E. (1977). A bromodeoxyuridine-mithramycin technique for detecting cycling and noncycling cells by flow microfluorometry. *Exp. Cell Res.,* **109,** 439.

VINDELΦV, L.L., CHRISTENSEN, I.J., JENSEN, G. & NISSEN, N.I. (1982*a*). Limits of detection of nuclear DNA abnormalities by flow cytometric DNA analysis. Results obtained by a set of methods for sample-storage, staining and internal standardization. *Cytometry,* **3,** 332.

VINDELΦV, L.L., CHRISTENSEN, I.J., KEIDING, N., SPANG-THOMSEN, M. & NISSEN, N.I. (1982*b*). Long-term storage of samples for flow cytometric DNA analysis. *Cytometry,* **3,** 317.

VINDELΦV, L.L., CHRISTENSEN, I.J. & NISSEN, N.I. (1982*c*). Standardization of high-resolution flow cytometric DNA analysis by the simultaneous use of chicken and trout red blood cells as internal reference standards. *Cytometry,* **3,** 328.

WINNEKER, R.C. & CLARK, J.H. (1983). Estrogenic stimulation of the antiestrogen specific binding site in rat uterus and liver. *Endocrinology,* **112,** 1910.